# A Synthetic *dl*-Nordihydroguaiaretic acid (Nordy), Inhibits Angiogenesis, Invasion and Proliferation of Glioma Stem Cells within a Zebrafish Xenotransplantation Model

**DOI:** 10.1371/journal.pone.0085759

**Published:** 2014-01-15

**Authors:** Xiaojun Yang, Wei Cui, Shicang Yu, Chuan Xu, Guilai Chen, Ai Gu, Tingting Li, Youhong Cui, Xia Zhang, Xiuwu Bian

**Affiliations:** 1 Key Laboratory of Tumor Immunopathology, Ministry of Education of China, Southwest Hospital, Third Military Medical University, Chongqing, China; 2 Institute of Pathology and Southwest Cancer Center, Southwest Hospital, Third Military Medical University, Chongqing, China; University of Bari Medical School, Italy

## Abstract

The zebrafish (*Danio rerio*) and their transparent embryos represent a promising model system in cancer research. Compared with other vertebrate model systems, we had previously shown that the zebrafish model provides many advantages over mouse or chicken models to study tumor invasion, angiogenesis, and tumorigenesis. In this study, we systematically investigated the biological features of glioma stem cells (GSCs) in a zebrafish model, such as tumor angiogenesis, invasion, and proliferation. We demonstrated that several verified anti-angiogenic agents inhibited angiogenesis that was induced by xenografted-GSCs. We next evaluated the effects of a synthetic dl-nordihydroguaiaretic acid compound (*dl*-NDGA or “Nordy”), which revealed anti-tumor activity against human GSCs *in vitro* by establishing parameters through studying its ability to suppress angiogenesis, tumor invasion, and proliferation. Furthermore, our results indicated that Nordy might inhibit GSCs invasion and proliferation through regulation of the arachidonate 5-lipoxygenase (Alox-5) pathway. Moreover, the combination of Nordy and a VEGF inhibitor exhibited an enhanced ability to suppress angiogenesis that was induced by GSCs. By contrast, even following treatment with 50 µM Nordy, there was no discernible effect on zebrafish embryonic development. Together, these results suggested efficacy and safety of using Nordy *in vivo*, and further demonstrated that this model should be suitable for studying GSCs and anti-GSC drug evaluation.

## Introduction

Glioblastoma is the most common primary adult brain tumor. The median survival time for glioblastoma remains less than 14 months after initial diagnosis, despite the improvement of therapeutic methods in recent years [1]. In this context, more evidence supports the notion that glioma stem cells (GSCs) are responsible for tumorigenesis, tumor cell invasion, angiogenesis, therapy resistance, and tumor recurrence [2]–[4]. Until now, several possible approaches to the therapeutic management of glioblastoma have been suggested. These include directly targeting GSCs by specific markers, disruption of the vascular niche that maintains GSCs, and titration of the developmental program that operates in GSCs to enforce their differentiation, due in part, to the enhanced sensitivity of their mature progeny to conventional therapy [5].

Zebrafish (*Danio rerio*) and their transparent embryos have recently emerged as another promising xenograft tumor model system in cancer research [6]. This model has shown many advantages including its simplicity for genetic manipulation, inexpensive maintenance, easy visualization of internal structures, and rapid embryonic development. Many reports have shown proliferation, angiogenesis, invasion and metastasis of human tumor cells within zebrafish embryos [7]–11. Our previous studies also indicated that the xenografted zebrafish embryonic tumor model is reliable for the evaluation of tumor-induced angiogenesis, invasion, and differentiation [12], [13]. However, whether zebrafish embryos would still remain useful for studying the biological features of GSCs is poorly studied. Recent reports have described the high-invasive capacity of xenografted-GSCs within this model [13], as compared with the low-invasive phenotype of differentiated U87 glioma cells. These observations encouraged us to study additional biological features of GSCs and to evaluate anti-GSC agents in this model.

Nordy (*dl*-nordihydroguaiaretic acid, *dl*-NDGA) is a synthetic compound that exhibits anti-tumor activity [14], [15]. Nordy triggered a differentiation program on glioma stem-like cells (GSLCs) towards an astrocytic phenotype that reduced the frequency of GSCs, and inhibited the growth of xenografted glioma cells by inhibiting the activity of Alox-5. These observations suggested the therapeutic potential of Nordy as an anti-GSC agent [16]. However, further evaluation of the therapeutic functions of Nordy in the settings of angiogenesis, tumor invasion, proliferation, and differentiation *in vivo* require further evaluation.

In this study, we investigated the utility of the zebrafish embryonic xenograft model for testing the effects of angiogenesis, proliferation and invasiveness induced by GSCs, and to evaluate the toxicity and anti-GSC capability of novel anti-cancer agents. We found that angiogenesis induced by xenografted human GSCs were significantly inhibited within Nordy-treated zebrafish embryos. Moreover, we showed that Nordy could suppress GSC invasion by promoting their differentiation in xenografted zebrafish embryos. In addition, compared with the Nordy-untreated control, GSC-xenografted zebrafish embryos showed that GSC proliferation was suppressed by Nordy treatment. Together, these observations suggest favorable efficacy and safety of Nordy, and further support the usefulness of zebrafish as a platform to study GSCs, and in evaluating the anti-GSC effect of candidate therapentic agents.

## Materials and Methods

### Ethics statement

This study was carried out in strict accordance with the recommendations in the Guide for the Care and Use of Laboratory Animals of the Third Military Medical University (TMMU). The protocol was approved by the Committee on the Ethics of Animal Experiments of Southwest Hospital, TMMU (No. 201110-1).

### Animal care and handling

Zebrafish (*Danio rerio*) and transgenic zebrafish Tg (*fli1*:EGFP)*^y1^*, which was a gift from Dr. Lu Wen, Sun Yat-Sen University, Guangzhou, China, were raised as previously described [17]. The fish were kept at 28°C in aquaria (ESEN Environ Science, China) with a 14 hrs light/10 hrs dark cycle. The embryos were raised at 28°C in E3 egg water until the desired developmental stages were achieved [18]. Embryos raised beyond 24 hrs post-fertilization (hpf) were treated with phenylthiourea (PTU; 0.003%, w/v; Sigma, USA).

### Cell culture and establishment of a stable red fluorescent protein (RFP) expressing glioma cell-line

All of the cell-lines were obtained from the American Type Culture Collection (ATCC, Manassas, VA, USA). U87 malignant glioma cells and Huh7 cells were maintained in Dulbecco's modified Eagle's medium (DMEM, Gibio, USA), and HCT116 cells were treated in RPMI-1640 cell culture medium (Gibio, USA), containing 10% fetal bevine serum (FBS) (Gibco, USA) and 1∶100 Pen/Strep (Invitrogen, USA). To establish the stable RFP-expressing cancer cell-line, cells were transfected with a pcDNA3.1 (+)-RFP vector by lipofectamine 2000™ (Invitrogen, USA). G-418 selections were performed at 48 hrs later. The stable RFP expressing cells were grown in corresponding cell culture medium containing 10% FBS, Pen/Strep antibiotics and G-418 (200 µg/ml; Invitrogen, USA).

### Isolation of GSCs from the enriched glioma sphere cells

We isolated GSCs from a human glioblastoma cell-line U87, and according to our previously described procedure [19]. Briefly, U87 cells were seeded in a 24-well plate at 2×10^4^ cells/well for 12–18 hrs. Thereafter, 250 µl culture medium was replaced with an equal volume of serum-free neural stem cell medium containing DMEM/F12 (Gibco, USA), B27 (Gibco, USA), recombinant human epidermal growth factor (rhEGF, 20 ng/ml; Sigma, USA), basic fibroblast growth factor (bFGF, 20 ng/ml; Upstate, USA), leukemia inhibitory factor (LIF, 10 ng/ml; Chemicon, USA), insulin (4 U/L; Sigma, USA), with/without vincristine (5 ng/ml; Hualian Pharmaceutical Co. China). This procedure was repeated every 24 hrs until several primary tumor spheres were visible by microscopy. At this point, the culture medium was removed and refilled with 1 ml fresh serum-free neural stem cell medium. The primary tumor spheres were dissociated and single cells were seeded into 24-well plates in a volume of 1 ml/well of serum-free neural stem cell medium. The culture medium was changed every 3 days. For isolating CD133-positive cells, U87 sphere cell suspensions were pelleted, and stained with anti-CD133/2 (293C3)-APC antibody (Miltenyi Biotech, Germany) for 30 min at 4°C. Next, CD133-positive cells were sorted by flow cytometry on the FACS Aria II (BD, USA). Staining with 7AAD (BD, USA) was used for flow cytometric exclusion of dead cells.

### Microinjection of human cancer cells

The cell transplantation protocol was modified from the previously published procedure [20]. The RFP expressing cells were washed, re-suspended in phosphate buffered saline (PBS, pH 7.0), and sorted by fluorescence-activated flow cytometry (FACS Aria II; BD, USA). Fluorescence was measured at a wavelength of 594 nm and fluorescence-emitting positive cells were collected in a fresh, sterile tube.

For tumor cells that were microinjected into embryos, zebrafish embryos were dechlorionated and anesthesized with tricaine (MS-222; Sigma, USA) 2 days post-fertilization (dpf) Tg (*fli1*:EGFP)*^y1^*. We adoptively transferred the desired cell numbers in the middle of the embryonic yolk *sac* using the Pneumatic Pico-Pump Injector (PLI-100; Harvard Apparatus, USA) with an injection needle (World Precision Instruments Inc., USA) drawn by a P-97 Flam/Brown Micropipette device (Sutter Instruments Co., USA). After injection, embryos were maintained for 1 hr at 28°C before incubation at 35°C. Embryos with fluorescent cells outside the desired injection region were excluded from further analysis.

### Whole mount immunofluorescence of zebrafish embryos

Angiogenesis and tumor invasion were evaluated as described previously [12], [13]. Briefly, after transplantation, the embryos were examined under an Olympus SZX-10 fluorescent microscope 2 days postinjection (dpi). All of the embryos were then mounted in 3% methylcellulose (Sigma, USA) so that they were oriented in the correct position for imaging. Both bright field and fluorescent images were captured with a QImaging digital camera controlled with Image-Pro Express software. Images were merged using an Adobe Photoshop CS2 (Adobe, USA) software program. The GFP labeled tumor angiogenesis and the relative emitted RFP fluorescence derived from adoptively transferred tumor cells were analyzed by ImageJ software (NIH, Bethesda, USA).

### VEGF Immunoassay

Approximately 1×10^5^ GSCs cells were seed into 24-well plates, and maintained in 0.5 ml DMEM cell culture medium with 0.5% FBS in each well. The tissue culture medium was collected at 24 hrs and 48 hrs, respectively. The VEGF^165^ concentrations were measured with the Human VEGF Quantikine ELISA Kit according to the supplied protocol (R&D System, USA).

### Quantitative real-time PCR (qRT-PCR)

Total RNA was extracted from tumor cells using TrizolTM Reagent (Invitrogen, USA) according to the manufacturer's protocol. The qRT-PCR assay was performed using SYBR PrimeScript RT-PCR Kit (TaKaRa, Japan) on a Rotor-Gene 6000 real-time genetic analyzer (Corbett Life Science, USA) according to manufacturer's instructions. The primer sequences of VEGF^165^ (GenBank: AB451322.1) and GAPDH as the internal control were: VEGF forward primer: 5′agccttgccttgctgctcta3′, reverse primer: 5′tttgatccgcataatctgca3′; GAPDH forward primer: 5′ tgcaccaccaactgcttagc3′, reverse primer: 5′ ggcatggactgtggtcatgag3′. The PCR protocol included a denaturation program (95°C for 2 min), followed by 40 cycles of amplification and quantification program (95°C for 5 sec, 55°C–57°C for 30 sec) and a melting curve program (55°C–95°C, with 0.5°C increments for each cycle). Each sample was replicated three times.

### Embryos treated with drugs and statistical analyses

The Nordy has been preserved in our lab [14], and Axitinib, Suntinib and Vatalanib were purchased from Selleck Company (USA). For Nordy treatment, the U87 cells were pre-treated with 50 µM Nordy before flow cytometric sorting and microinjection. All of the compounds were then dissolved in 1% DMSO and added into E3 embryo medium at 2 dpf after microinjection with a corresponding final concentration, which did not appreciably affect native zebrafish embryonic development. All the microinjected Tg (*fli1*:EGFP)*^y1^* zebrafish embryos with/without drug treatment were captured at the desired stages. All results are presented as arithmetic mean ± SEM, which were analyzed by SPSS10.0 statistical software. Statistical analyses was performed by Student's *t*-test.

## Results

### Anti-tumor drug evaluation in a GSC-xenografted zebrafish embryos model

**Figure 1:**
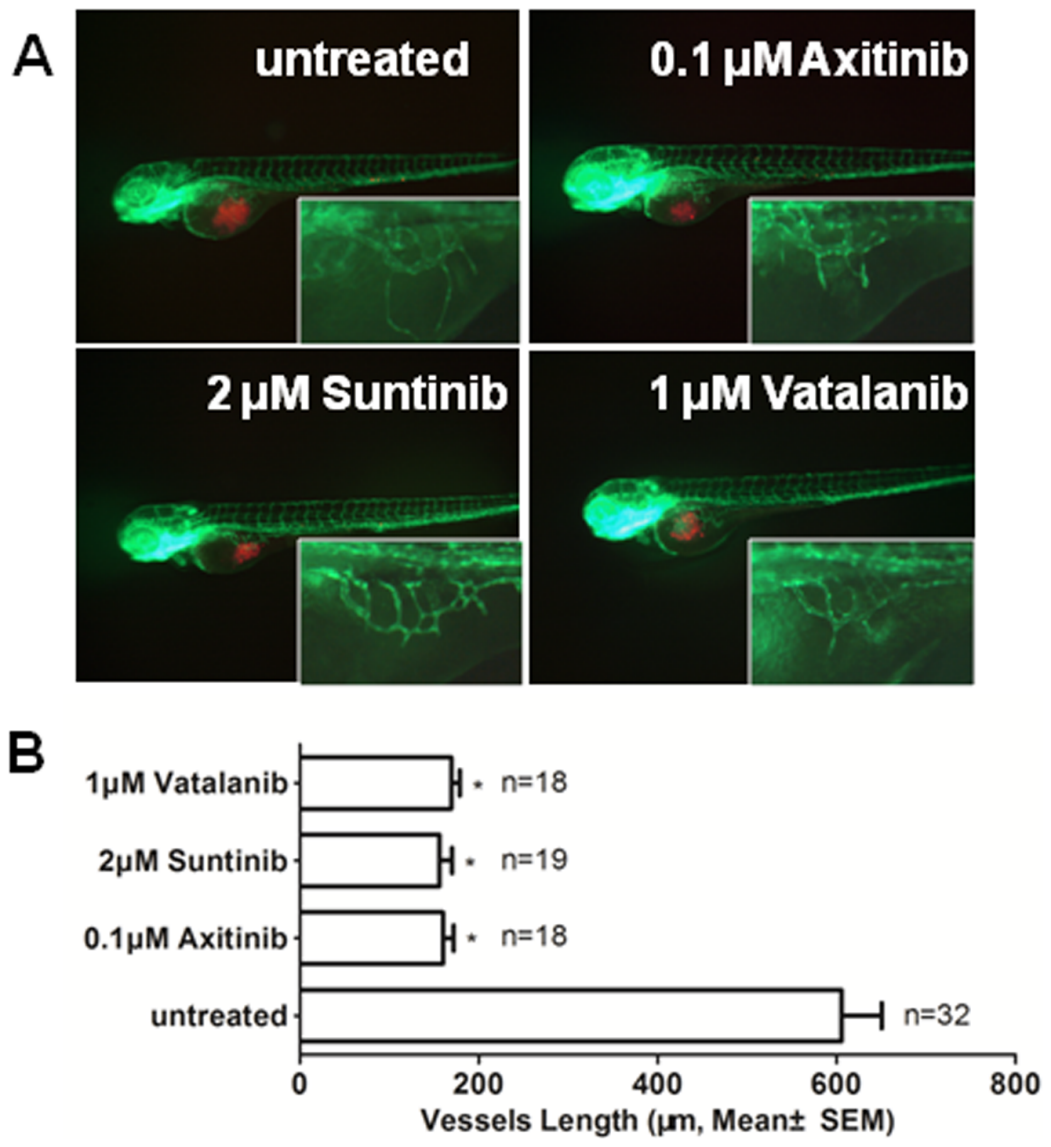
The effects of verified VEGF receptor tyrosine kinase inhibitor on angiogenesis that was induced by GSCs within zebrafish embryos. A. The representative images of the inhibition of angiogenesis with axitinib, suntinib and vatalanib treatment. B. Quantitative analysis of the length of newly formed vessels that were induced by U87 GSCs with/without axitinib, suntinib and vatalanib treatment at 2 dpi. (P<0.0001)

Based on previous reports, the xenografted tumor cells in the zebrafish embryo have attracted considerable attention in recent years as a model system for cancer research [7], [8], [10], [21]. Our previous study also demonstrated that certain unique features of GSCs could be studied with this model [13]. We then extended it as a reliable system for GSCs research of tumor angiogenesis, invasion, and proliferation, and in the evaluation of certain candidate anti-GSC agents. To determine whether this model was suitable for the angiogenesis induced by GSCs, we first measured tumor angiogenesis induced by U87 GSCs and the angiogenic inhibition by several verified VEGF receptor tyrosine kinase inhibitors, such as Axitinib, Suntinib and Vatalanib [22]–[27]. To explore the suitable drug concentration, which does not appreciably affect embryonic vascular development, including intersegmental blood vessel disruption and/or subintestinal vein developmental (arrows, Figure S1), we assessed the survival and morphology of embryos treated with all three compounds (Table S1). As expected, all compounds exhibited significant inhibition of angiogenesis that was induced by xenografted U87 GSCs (Figure 1). These results showed that the xenografted zebrafish embryonic model is sensitive and reliable for GSCs-induced angiogenesis research.

### The normal developmental responses of zebrafish embryos to Nordy treatment

**Figure 2:**
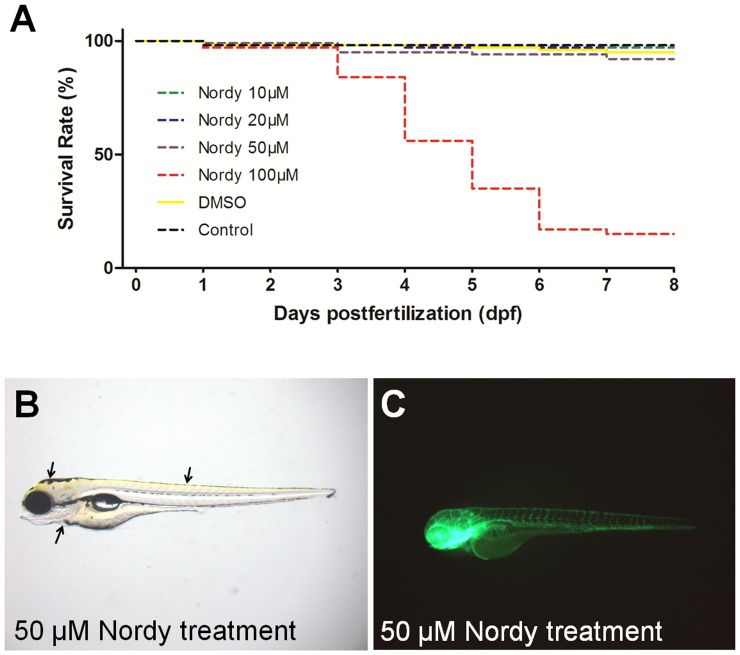
The response to Nordy treatment on the survival rate, and development of zebrafish embryos. A. Quantitative analysis of the survival rate of naïve zebrafish embryos following treatment with various concentrations of Nordy. B. Showing the representative bright field images of native zebrafish embryos that were incubated with 50 µM Nordy. C. The representative images of vascular development of Tg (*fli1*:EGFP)*^y1^* embryos following treatment with 50 µM Nordy.

Based on our results, we next evaluated the potential anti-tumor effects of Nordy, which was found to have anti-GSC effects by promoting GSC differentiation [16], and for its activity on angiogenesis, tumor invasion, and proliferation. Our previous work indicated that the concentration of Nordy treatment for U87 glioma cell-line varied from 10 µM to 100 µM *in vitro* [16]. Therefore, similar studies were done for testing the toxicity of Nordy on the normal embryonic development of zebrafish. The embryos (n = 100/group) were incubated with different concentrations (10 µM, 20 µM, 50 µM and 100 µM) of Nordy in E3 embryonic medium at the one-cell embryonic stage. The embryos were then observed throughout the first 8 dpf of life for survival and morphology. At 8 dpf the survival rate indicated that 50 µM Nodry did not lead to higher mortality (8 dead in 100 embryos) as compared with the DMSO vehicle control group (5 dead in 100 embryos). However, 100 µM Nodry treatment was associated with a very high embryonic mortality rate (over 40% at 4 dpf and 80% at 8 dpf), suggesting that a dose of 50 µM Nodry should be suitable for the following experiments (Figure 2A). As shown in Figure 2B and Figure S2, the morphology of zebrafish embryos that had been incubated with 10 µM or 50 µM Nodry was similar to the morphology of normal embryos. The development of the major organs including the heart, brain, and neural tube revealed no discernible defects at 4 dpf (arrows, Figure 2B). Further analysis was performed to determinate whether vascular development could be disrupted by Nordy treatment. We also found that there was also no appreciable disruption in vascular development,or embryonic development, in 50 µM Nordy treated Tg (*fli1*:EGFP)*^y1^* embryos at 4 dpf (Figure 2C). These observations indicated that even 50 µM Nordy treatment did not appreciably affect embryonic development of native zebrafish.

### Nordy inhibits GSC-induced angiogenesis within zebrafish embryos

**Figure 3:**
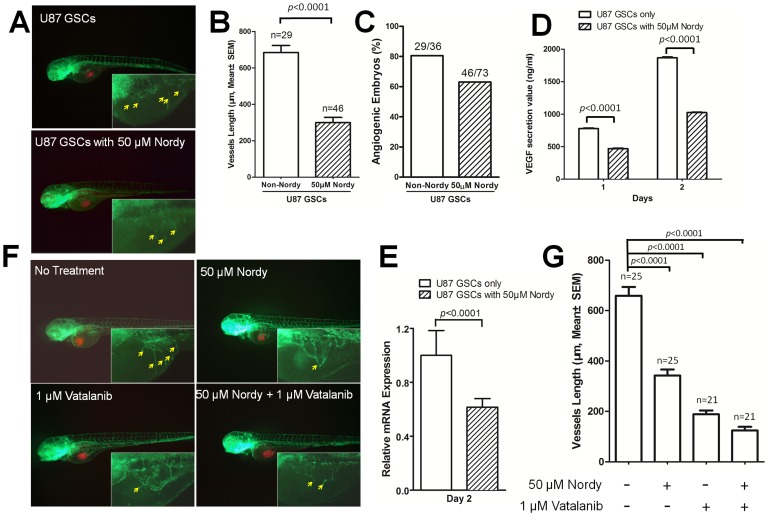
Analysis of angiogenesis induced by U87 GSCs with/without Nordy treatment. A. The representative merged images of angiogenesis as induced by U87 GSCs with/without Nordy treatment. The images at higher magnification show the new vessels that were induced by U87 GSCs. B. Quantitative analysis of the length of newly formed vessels that were induced by U87 GSCs with/without Nordy treatment at 2 dpi. C. Quantitative analysis of the percentage of angiogenic embryos with/without Nordy treatment at 2 dpi. D. Showing the secreted VEGF^165^ volume of U87 GSCs with/without Nordy treatment *in vitro*. E. Showing the VEGF^165^ mRNA level in U87 GSCs with/without Nordy treatment as assayed by qRT-PCR at 2 dpi. F. Showing the representative merged images of angiogenesis that were induced by U87 GSCs treated with/without Nordy and/or vatalanib. The images at higher magnification showed new vessels induced by tumor cells. G. Showing quantitative analysis of the length of newly formed vessels induced by U87 GSCs with/without Nordy and/or Vatalanib treatment. The yellow arrow indicates newly formed angiogenic vessels on embryonic yolk *sac* ball. Red: injected RFP-labeled U87 GSCs cells; green: GFP fluorescence of vasculature in Tg (*fli1*:EGFP)*^y1^* embryos.

Our previous describe demonstrated angiogenic phenotype induced by U87 differentiated cells [12]. We then evaluated the angiogenesis induced by U87 GSCs within this model. As we expected, there are also similar angiogenic phenotype after GSCs injection in zebrafish (Figure S3). Since the verified VEGF receptor tyrosine kinase inhibitors markedly inhibit angiogenesis induced by xenografted U87 GSCs, we next evaluated the effects of Nordy, which is also capable of modulating several hallmarks of cancer including robust proliferation, and resistance to apoptosis by inhibiting the Alox-5 pathway in the process of tumor angiogenesis *in vivo* [28]–[30]. Since Nordy regulates VEGF expression levels in human glioma cells *in vitro* [31], [32], we hypothesized that it might play a role in the inhibition of GSCs-induced angiogenesis. According to our previous studies [12], we analyzed the length of newly formed vessels induced by U87 GSCs and the frequency of angiogenic embryos with/without 50 µM Nordy treatment (Figure 3B and 3C). We found that Nordy markedly inhibited angiogenesis that was induced by U87 GSCs (Figure 3A). Quantitative analyses indicated that the newly formed vascular length that was induced by U87 GSCs was inhibited by 54% (Figure 3B).

The induction of new vessels was involved in the development of host vasculature and/or tumor angiogenesis, and the apparent disruption of normal embryonic vasculature development was undetectable with 50 µM Nordy treatment (Figure 2C). Besides, the induced new vessels did not physically interact with the xenografted tumor cell mass (data not shown). We expected that the VEGF^165^ sectioned from tumor cells might be involved in angiogenesis. We next examined VEGF^165^ secretion in U87 GSCs cells with/without Nordy treatment *in vitro*. VEGF immunoassay and qRT-PCR analysis revealed that Nordy treatment remarkably suppressed secreted VEGF^165^ levels by U87 GSCs (Figure 3D and 3E). Therefore, we inferred that the inhibition of GSC-induced angiogenesis by Nordy was also involved in the suppression of VEGF secretion from xenografted GSCs within zebrafish embryos.

Since Nordy can inhibit angiogenesis by regulating CXCR4-mediated production of VEGF by glioma cells [32], we attempted to confirm whether the combination of Nordy and the VEGF receptor tyrosine kinase inhibitor could enhance suppression of angiogenesis that was induced by GSCs (Figure 3F). Inhibition of angiogenesis by both Nordy and Vatalanib (19%) was more significant than that found for either of them used alone (52% and 28.6%, Figure 3G). Also, pre-treatment of Huh7 cells and HCT116 cells xenografted into embryos with Nordy and/or Vatalanib, showed similar morphologic phenotypes (data not shown).

### Nordy inhibits GSCs invasion through promoting the differentiation of GSCs

**Figure 4:**
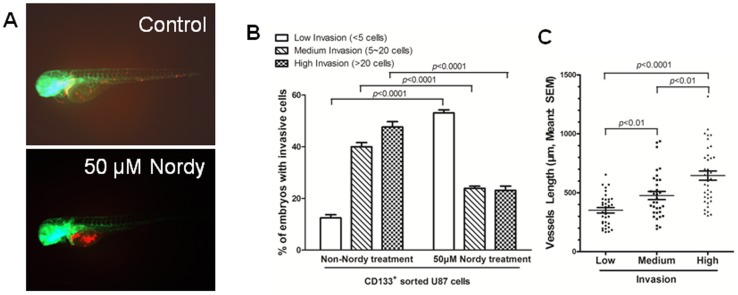
CD133 positive U87 GSCs were suppressed by Nordy treatment within zebrafish embryos. A. The representative high-invasion phenotypes of injected GSCs with/without Nordy treatment. B. The inhibitory effect of Nordy on the invasion of CD133 positive U87 GSCs within zebrafish embryos. The percentages of invasive cells in adoptively transferred embryos (low, medium, or high-invasion) were measured at 2 dpi. The data were obtained from three replicate experiments with the number of embryos: n = 120 for the non-Nordy treated group, n = 113 for the Nordy treated group. C. The relationship between angiogenesis induced by GSCs and degree of invasion. N = 34 represented the low-invasion group, n = 33 represented the medium-invasion group, and n = 39 represented the high-invasion group. Red: adoptively transferred RFP-labeled U87 GSCs cells; green: represents the GFP fluorescence of angiogenesis in Tg (*fli1*:EGFP)*^y1^* embryos.

Our previous study indicated that Nordy induces differentiation and inhibits self-renewal of GSLCs *in vitro* [16]. Meanwhile, compared with low-invasive feature of differentiated U87 cells, GSCs exhibits a highly invasive phenotype within zebrafish embryos [13]. We therefore examined the effects of Nordy on GSC invasion in U87 GSCs-xenografted zebrafish embryos. We treated the embryos with 50 µM Nordy after microinjection in the middle of the yolk *sac*, and then examined the invasiveness of the tumor at 2 dpi. According to the classification of the degrees of invasion [10], [13]: Low: less than 5 migrated cells; Medium: 5–20 migrated cells, and High: more than 20 migrated cells, the invasion of U87 GSCs within the embryos was significantly inhibited by 50 µM Nordy treatment (Figure 4A and Figure S4). The percentage of embryos demonstrated medium- and high-invasive phenotypes with the treatment of 50 µM Nordy were 23.9% and 23.1% as compared with approximately 40% and 47.6% within non-Nordy treated embryos, respectively (Figure 4B and Table 1). We also noted that Nordy treatment promoted CD133 positive glioma cell differentiation *in vitro* [16]. In addition, our previous studies demonstrated that GSCs, which can be regarded as CD133 positive cells, demonstrated a higher invasive phenotype in this model. By contrast, CD133 negative U87 glioma cells (also regarded as U87 differentiated glioma cells) almost failed to migrate from the original injection site to remote region, suggesting a low-invasive phenotype of glioma differentiated cells in zebrafish embryos [13]. Thus, the positive correlation between GSCs invasion and the induction of GSCs differentiation indicated that Nordy inhibited GSCs invasion *in vivo*.

**Table 1:** Quantitation of invading U87 CD133^+^ GSCs within zebrafish embryos injected with/without 50 µM Nordy treatment at 2 dpi.

Groups (No. of injected cells)	Total no. of injected embryos	No. of injected embryos with invasive cells (mean ± SD)			Dead
		Low-invasion	Medium-invasion	High-invasion	
Non-Nordy treated group (n = 300)	150	12.5±4.8%	40±11%	47.5±15.8%	30
Nordy treated group (n = 300)	150	53±9%	23.9±4.4%	23.1±8.5%	37

The data were obtained from three replicate experiments of 50 injected embryos for each experiment.

Moreover, both the length of newly formed vessels that were induced by GSCs, and the extent of invasion in the same GSCs-injected embryos were measured to reveal the relationship between GSCs-induced angiogenesis and extent of invasion. We found a positive correlation between angiogenesis and the extent of tumor invasion. Notably, the newly formed vessel lengths induced by GSCs in the low-, medium-, and high-invasion groups were 334 µm, 476 µm, and 633 µm, respectively, which is consistent with our previous work that described the process of GSCs invasion in a zebrafish model [13]. All these results suggested that GSCs can induce newly formed vessels that results in a higher invasive phenotype after microinjection of GSCs through more newly formed vasculature within zebrafish embryos.

### The proliferation of GSCs were suppressed by Nordy treatment within zebrafish embryos

**Figure 5:**
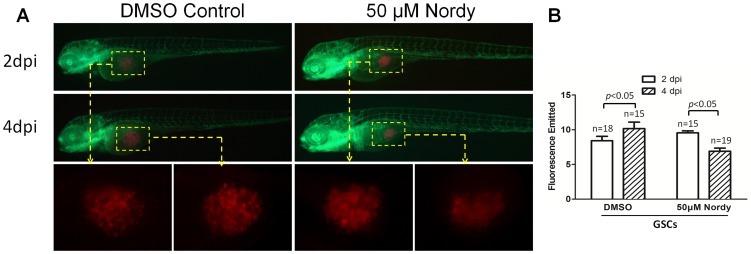
Effects of Nordy on the proliferation of GSCs within zebrafish embryos. A. Representative merged images of GSC proliferation with/without Nordy treatment within zebrafish embryos. B. Quantitative analysis of the emitted fluorescence of RFP-labeled GSCs with/without Nordy treatment at 2 dpi and 4 dpi. At higher magnification, the images showed the injected the RFP labeled GSCs accumulate within the embryos (yellow broken box). Red: injected U87-RFP cells; green: GFP fluorescence of angiogenesis in Tg (*fli1*:EGFP)*^y1^* embryos.

We performed microinjection in the mid yolk *sac* and analyzed the emitted fluorescence to examine the proliferation of RFP-labeled GSCs, according to a previous modified protocol [7], [12]. We found that the proliferation of U87 GSCs was not apparent even at 4 dpi within zebrafish embryos (data not shown). However, U87 GSCs mixed with Matrigel I (BD, USA) exhibited obvious proliferation in the middle of the yolk *sac* at 4 dpi. We therefore determined whether Nordy treatment could affect the proliferation of U87 cells in this model. After microinjecting approximately 300 sorted CD133-expressing U87 cells into the embryos, 50 µM Nordy were then added to the E3 embryonic medium. The same embryo was observed at 2 dpi and 4 dpi under the same observation conditions (Figure 5A). The fluorescence emitted by each group within the embryos was quantitated by ImageJ software (Figure 5B). Determination of the emitted fluorescence indicated a reduction in the number of injected GSCs in zebrafish at 4 dpi, whereas the proliferation of RFP-labeled U87 GSCs appeared unaffected (Figure 5B). Also, our previous work indicated that Nordy-treated GSLCs did not show an appreciable difference in apoptosis compared to non-treated controls [16]. We expected that this reduction in the numbers of injected GSCs was induced by physical injury of injected GSCs after flow cytometric sorting, and decreased Nordy treatment for GSC proliferation.

In summary, these results indicated that this GSCs-xenografted zebrafish model is reliable for the evaluation of an anti-tumor compound. Nordy treatment could block angiogenesis, tumor invasion, and proliferation in the GSC-xenografted zebrafish model, which is consistent with our previous studies *in vitro* [14]–16.

## Discussion

Accumulating evidence is emerging that informs us that tumors contain a very small subpopulation of cancer stem cells (CSCs). They possess a variety of unique biologic properties including self-renewal, propagation and production of differentiated progeny, expression of specific cell surface markers, and which are regarded as the root cause of tumorigenesis, tumor invasion, angiogenesis, and therapy resistance. Although the mouse has long remained the model of choice for cancer research and drug screening [33], there are several inherent disadvantages associated with this xenografted model, as compared with the promising approach of using the zebrafish xenografted model.

As a vertebrate model system, the zebrafish is completely transparent, facilitating *in vivo* observations. The interaction of GSCs and the host microenvironment, such as zebrafish cells, organs, and vasculature, can be observed clearly under the fluorescent microscope at an early stage. We can also easily track the migration and differentiation of specific marker labeled GSCs, even at the individual cancer cell level in a whole xenografted animal. Furthermore, the rapid development of GSCs allows visualization of major organs in the first few days of life, which greatly facilitates experimental throughput.

Our results demonstrated that the observation period for studies of angiogenesis, tumor invasion and GSCs proliferation in zebrafish embryos is within 3 to 4 days of development, which is much shorter than the mouse model, which is in the order of several weeks to months. An additional disadvantage of using the mouse model is that a large number of cells (approximately 1×10^6^) are required. In the zebrafish model, fewer injected GSCs (even less than 100 cells per embryo) can induce significant angiogenic phenotypes, which is particularly suitable for GSC angiogenic research because of the difficulty in isolating GSCs through flow cytometric sorting (e.g., less than 1% in the U87 glioma cell-line).

Due to the permeability and aqueous environment of zebrafish embryos, candidates of anti-tumor drugs can be delivered directly to the fish water to assess their cytotoxic, apoptotic, or anti-angiogenic effects. In addition, compared to studies using mice, the zebrafish platform is more convenient in terms of quantitative microplate analyses and image analysis for *in vivo* studies and high-throughput screening. Moreover, the effects of treatment are readily detectable in this system within days following treatment, suggesting greater economic benefits and high-throughput applications for screening anti-tumor agents. Thus, studies of the efficacy and safety of anti-GSC agents on glioblastoma would aid in the development of clinical anti-tumor strategies.

Previous efforts have described the utility of zebrafish embryos for investigating angiogenesis, tumor invasion and metastasis, and high-throughput anti-tumor screening [6]. In this work we have extended the usefulness of zebrafish for studying GSC features and the systematic evaluation of the anti-GSC agent Nordy. We found significant angiogenic phenotypes through tumor angiogenesis factors (e.g., VEGF, SDF-1) secreted from the xenografted GSCs within zebrafish embryos. Inhibition of angiogenesis that was induced by U87 GSCs following treatment with several verified VEGF receptor tyrosine kinase inhibitors revealed the reliability of Nordy as an anti-angiogenic agent. Nordy also demonstrated anti-angiogenic effects by inhibiting the Alox-5 pathway [16]. Although inhibition of GSCs proliferation by Nordy might also result in dampening of angiogenesis, we expected that Nordy's primary anti-angiogenic effect is its ability to significantly inhibit the expression of secreted VEGF. This was evident because the qRT-PCR assay showed that VEGF expression of the same cell number was significantly decreased following Nordy treatment *in vitr*o (Figure 3D and 3E). We also noticed that at higher drug concentrations Nordy displayed lower inhibition of angiogenesis that was induced by GSCs as compared with treatment by Vatalanib. Our findings indicated that Nordy treatment might enhance the anti-angiogenic effect of Vatalanib within safe drug treatment limits because of their different target effects [23], [32], which suggests its potential value as a clinical anti-angiogenic strategy.

As we know, GSCs exhibited a much higher invasive capability within zebrafish embryos [2]–[4], [13]. Nordy is also an anti-GLSC agent since it regulates differentiation and growth inhibition by inhibiting the Alox-5 pathway [16]. This was associated with carcinogenesis, robust proliferation, and tissue invasion [28]–. The transparency of the zebrafish facilitates detection of suppressed GSC invasion following Nordy treatment. Wang *et al.* [16] examined the frequency of CD133 positive cells in GSLCs with/without 50 µM Nordy treatment by flow cytometry. The percentage of CD133 positive cells decreased from 6.47% to 0.9% following Nordy treatment. Therefore, inhibition of GSCs and especially Nordy-mediated inhibition of the medium- and high-invasiveness may be associated with its ability to promote GSC differentiation *via* regulation of the Alox-5 pathway (Figure 4B and Table 1). In addition, the positive correlation between angiogenesis and invasion induced by GSCs within zebrafish embryos suggested that more newly formed vessels may offer a convenient route for GSCs invasion *in vivo*. This is similar to the phenotypes seen in our previously described work [34]. Furthermore, suppression of angiogenesis by Nordy might in turn inhibit GSC invasion.

It is interesting to compare the proliferation of various tumor cells within zebrafish embryos. Previous reports of transplanted glioma U251 cells and melanoma cells in zebrafish, demonstrated an obvious proliferation phenotype [7], [8]. By contrast, in U87 cells [12], [13], we did not find any apparent proliferation after microinjection into the embryonic yolk *sac* at 2 dpi. The proliferation of tumor cells may result from different cellular properties and methodology, such as the microinjection site. We noticed that U251 cells or melanoma cells were injected directly into embryonic tissues or had migrated to the host tissue from the original injection site. In our study, because of the higher embryonic mortality rate and fewer injected tumor cells into the host tissues, we performed microinjection to the mid yolk *sac*, which might be different from the real tumor microenvironment. Alternatively, the Matrigel-mixed GSCs exhibited a recognizable proliferative phenotype, yet their higher-invasive capability was markedly inhibited (Figure 4 and Figure 5). We expected that on the one hand Matrigel-I might offer higher levels of essential ingredients that would support proliferation of xenografted U87 GSCs in the mid yolk *sac*. By contrast, Matrigel-I remarkably promotes GSC differentiation and enhances the adhesion of the injected-GSCs at an early stage, which inhibits the subsequent invasive capability of U87 GSCs. Nevertheless, Nordy treatment leads to obvious suppression of GSCs proliferation. Since high-levels of arachidonic acid are associated with promoting cancer cell proliferation in breast cancer tissues [35], we speculated that inhibition by Nordy may also be involved in regulating the Alox-5 pathway.

In summary, our results suggest that this improved GSC-xenografted zebrafish model could provide a potential economically viable tool to study features of GSCs, and evaluate the efficacy and safety of specific anti-GSC agents. Moreover, our study suggests that GSCs might serve as a potential bridge to resource- and time-intensive studies in animal models, and ultimately to clinical trials.

## Supporting Information

Figure S1 **Vascular disruption phenotypes.** A. Showing intersegmental blood vessel disruption. B. Showing subintestinal vein developmental disruption.
(TIF)

Figure S2 **The morphology of zebrafish embryos incubated with 10 µM, and 50 µM Nodry as compared to the morphology of normal embryos.**
(TIF)

Figure S3 **Angiogenesis induced by differentiated U87 cells and U87 in GSC in zebrafish.** A: Representative merged images of angiogenesis induced by differentiated U87 cells and U87 GSCs in zebrafish embryos. The images here are at a higher magnification and showed new vessels that were induced by tumor cells. B. Quantitative analysis of the length of newly formed vessels induced by differentiated U87 cells and U87 GSCs in zebrafish embryos. C. Quantitative analysis of the percentage of angiogenic embryos induced by differentiated U87 cells and U87 GSCs.
(TIF)

Figure S4 **Time-lapse merged images of invasive U87 GSCs with/without Nordy treatment within zebrafish embryos (6 hpi, 18 hpi, 30 hpi, 42 hpi, and 54 hpi).**
(TIF)

Table S1 **The phenotypes of verified VEGF receptor tyrosinee kinase inhibitors at different concentrations for the development of normal zebrafish embryos.**
(DOC)
