# Supplementary figures and images for: A Synthetic dl-Nordihydroguaiaretic acid (Nordy), Inhibits Angiogenesis, Invasion and Proliferation of Glioma Stem Cells within a Zebrafish Xenotransplantation Model
Source: PLoS One. 2014 Jan 15;9(1):e85759. doi: 10.1371/journal.pone.0085759 (PMC3893259; doi:10.1371/journal.pone.0085759)

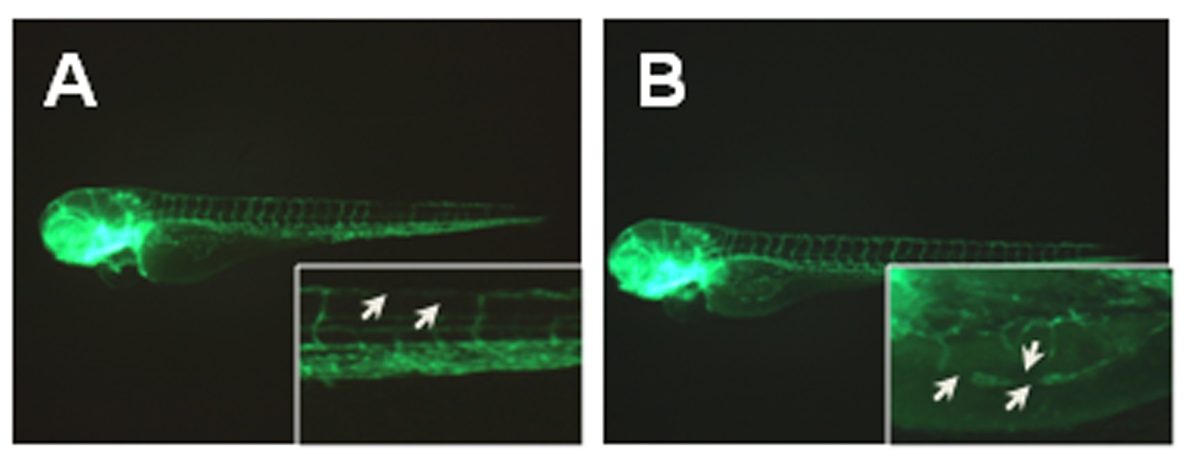

Supplement: Figure S1 — Vascular disruption phenotypes. A. Showing intersegmental blood vessel disruption. B. Showing subintestinal vein developmental disruption. (TIF) [file pone.0085759.s001.tif]

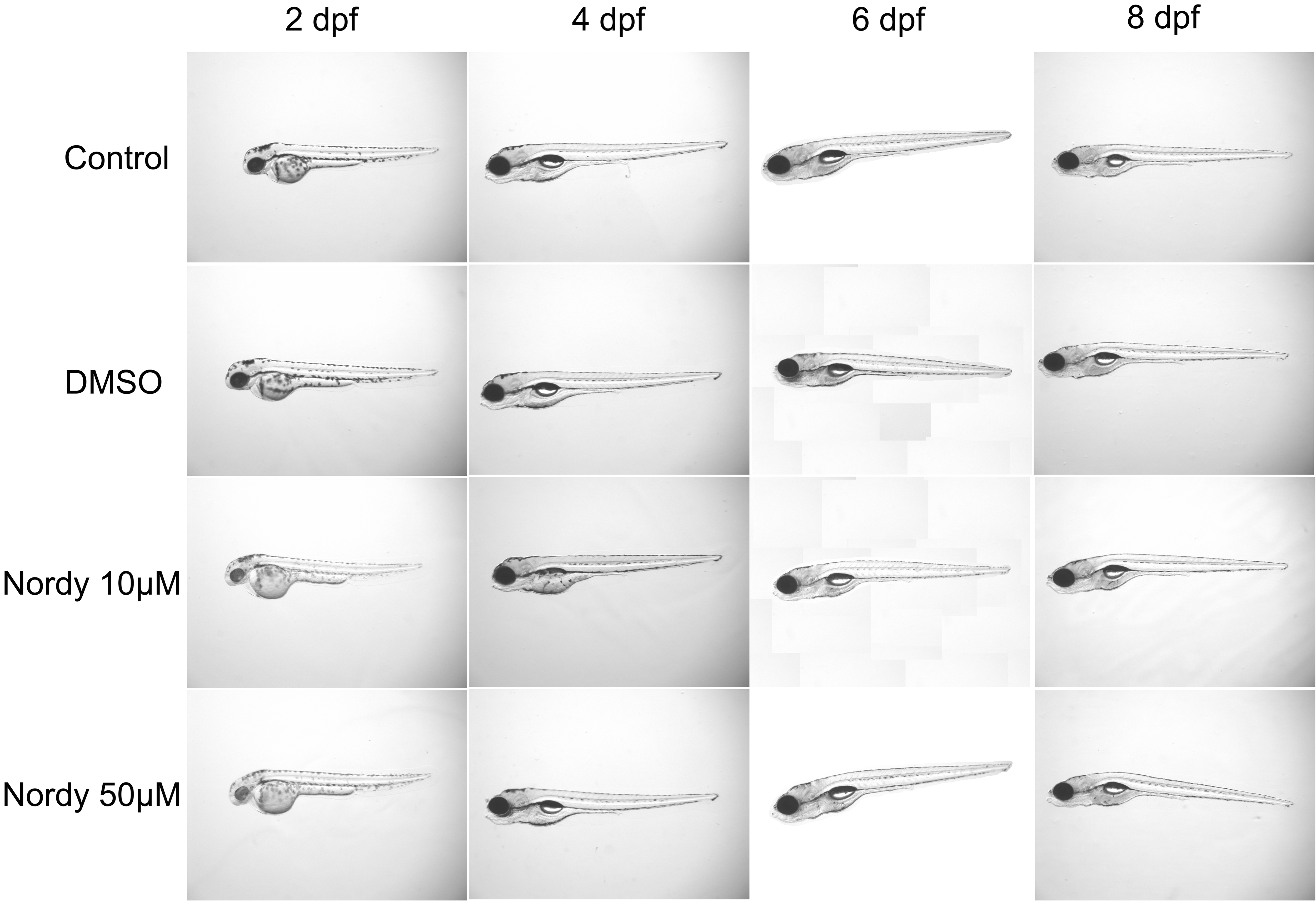

Supplement: Figure S2 — The morphology of zebrafish embryos incubated with 10 µM, and 50 µM Nodry as compared to the morphology of normal embryos. (TIF) [file pone.0085759.s002.tif]

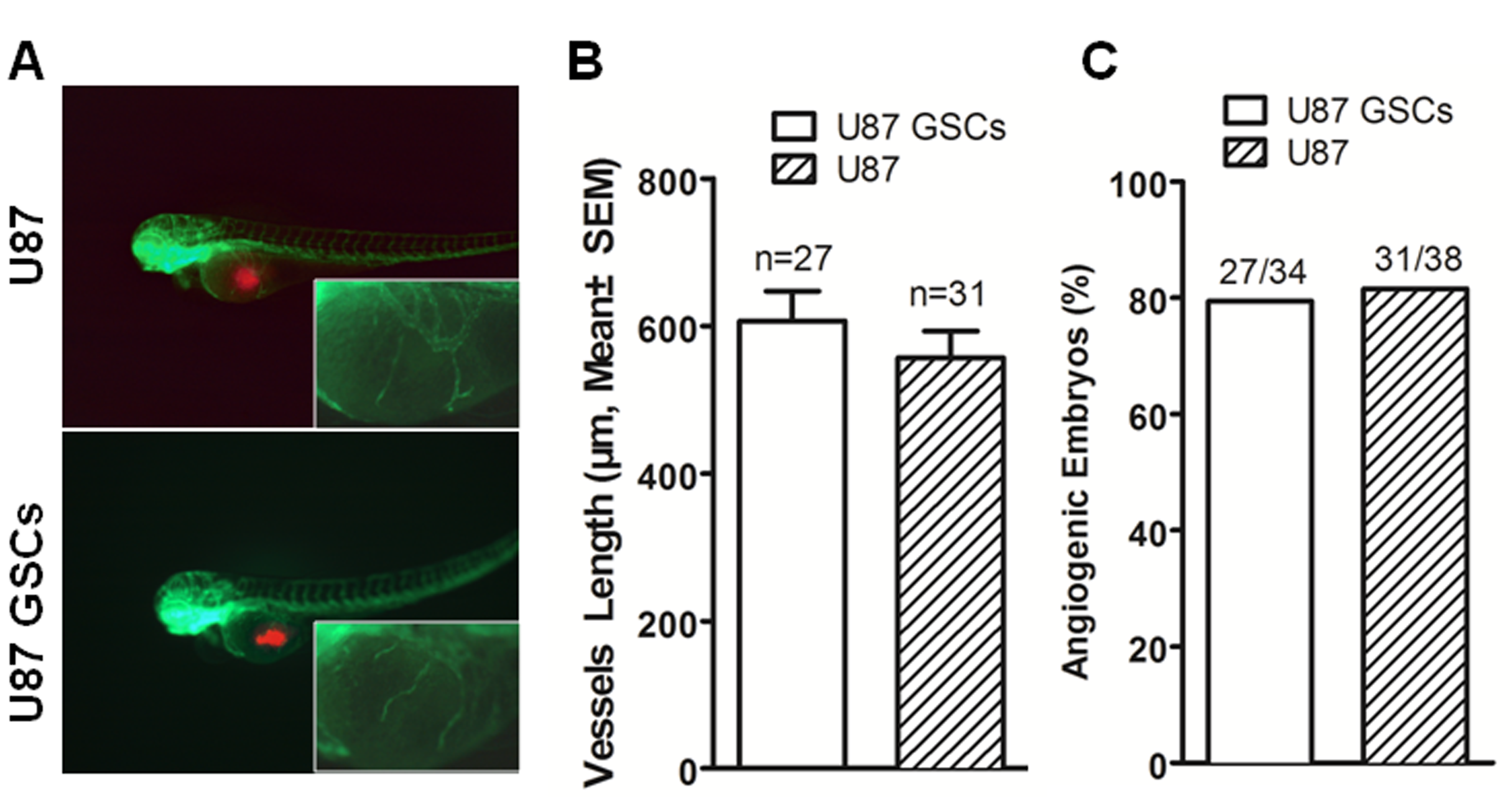

Supplement: Figure S3 — Angiogenesis induced by differentiated U87 cells and U87 in GSC in zebrafish. A: Representative merged images of angiogenesis induced by differentiated U87 cells and U87 GSCs in zebrafish embryos. The images here are at a higher magnification and showed new vessels that were induced by tumor cells. B. Quantitative analysis of the length of newly formed vessels induced by differentiated U87 cells and U87 GSCs in zebrafish embryos. C. Quantitative analysis of the percentage of angiogenic embryos induced by differentiated U87 cells and U87 GSCs. (TIF) [file pone.0085759.s003.tif]

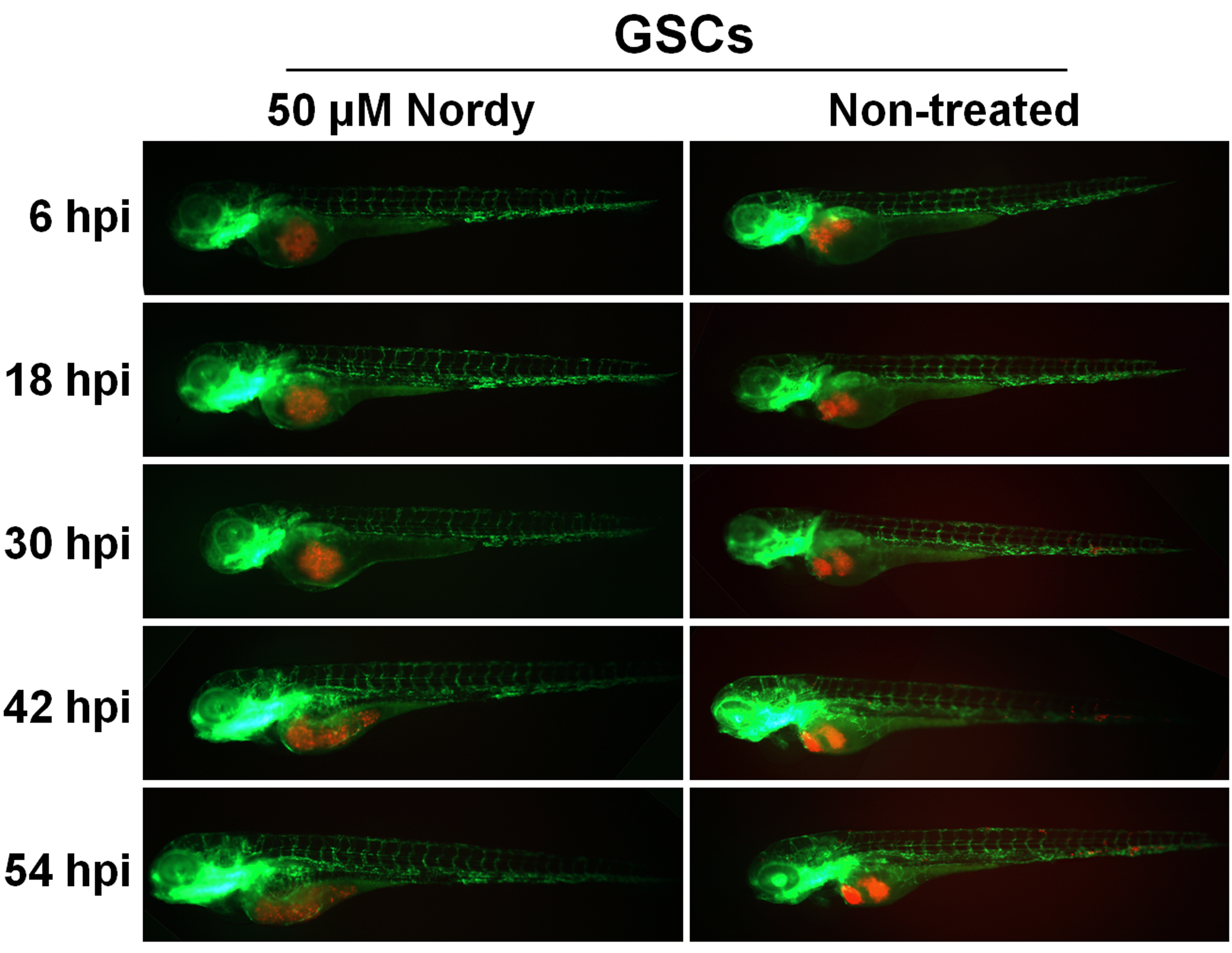

Supplement: Figure S4 — Time-lapse merged images of invasive U87 GSCs with/without Nordy treatment within zebrafish embryos (6 hpi, 18 hpi, 30 hpi, 42 hpi, and 54 hpi). (TIF) [file pone.0085759.s004.tif]
