# Supplementary material for: A Synthetic dl-Nordihydroguaiaretic acid (Nordy), Inhibits Angiogenesis, Invasion and Proliferation of Glioma Stem Cells within a Zebrafish Xenotransplantation Model
Source: PLoS One. 2014 Jan 15;9(1):e85759. doi: 10.1371/journal.pone.0085759 (PMC3893259; doi:10.1371/journal.pone.0085759)
Supplement: Table S1 — The phenotypes of verified VEGF receptor tyrosinee kinase inhibitors at different concentrations for the development of normal zebrafish embryos. (DOC) [file pone.0085759.s005.doc]

| **Name** | **Concentration (μM)** | **Phenotypes** | | | |
| --- | --- | --- | --- | --- | --- |
|  |  | **Death** | **Intersegmental blood vessel disruption** | **Subintestinal veins developmental defect** | **Normal** |
| **Axitinib** | 10 | 0/18 | 17/18 | 16/18 | 0/18 |
|  | 5 | 0/19 | 18/19 | 18/19 | 0/19 |
|  | 2 | 0/19 | 14/19 | 19/19 | 0/19 |
|  | 1 | 0/21 | 10/21 | 21/21 | 0/21 |
|  | 0.5 | 0/18 | 5/18 | 16/18 | 2/18 |
|  | 0.2 | 0/19 | 0/19 | 11/19 | 8/19 |
|  | 0.1 | 0/23 | 0/23 | 3/23 | 20/23 |
|  | 0.05 | 0/22 | 0/22 | 0/22 | 22/22 |
| **Suntinib** | 200 | 10/10 | 0/10 | 0/10 | 0/10 |
|  | 100 | 0/10 | 10/10 | 10/10 | 0/10 |
|  | 50 | 0/10 | 8/10 | 9/10 | 0/10 |
|  | 20 | 0/10 | 0/10 | 8/10 | 2/10 |
|  | 10 | 0/10 | 2/10 | 8/10 | 0/10 |
|  | 5 | 0/11 | 1/11 | 3/11 | 8/11 |
|  | 2 | 0/11 | 0/11 | 0/11 | 11/11 |
|  | 1 | 0/12 | 0/12 | 0/12 | 12/12 |
| **Vatalanib** | 200 | 10/10 | 0/10 | 0/10 | 0/10 |
|  | 100 | 0/13 | 12/13 | 12/13 | 0/13 |
|  | 50 | 0/15 | 13/15 | 13/15 | 0/15 |
|  | 20 | 0/14 | 5/14 | 10/14 | 2/14 |
|  | 10 | 0/12 | 2/12 | 11/12 | 1/12 |
|  | 5 | 0/11 | 0/11 | 8/11 | 3/11 |
|  | 2 | 0/10 | 0/10 | 6/10 | 4/10 |
|  | 1 | 0/14 | 0/14 | 0/14 | 14/14 |
